# The contraceptive efficacy of intravas injection of Vasalgel™ for adult male rhesus monkeys

**DOI:** 10.1186/s12610-017-0048-9

**Published:** 2017-02-07

**Authors:** Angela Colagross-Schouten, Marie-Josee Lemoy, Rebekah I. Keesler, Elaine Lissner, Catherine A. VandeVoort

**Affiliations:** 1 California National Primate Research, University of California, One Shields Ave, Davis, CA 95616 USA; 2 Department of Obstetrics and Gynecology, University of California, Davis, CA 95616 USA; 3 Parsemus Foundation, PO Box 2246, Berkeley, CA 94702 USA

**Keywords:** Primate, *Macaca mulatta*, Hydrogel, Vas deferens, Styrene maleic acid polymer, Fertility, Vasectomy, Vasalgel, Contraception, Sterilization, Colony management, Primate, *Macaca mulatta*, Hydrogel, Canal déférent, Polymère d’acide styrène-alt-maléique, Fécondité, Vasectomie, Vasalgel™, Contraception, Stérilisation, Gestion de colonie

## Abstract

**Background:**

Options for male contraception are limited. The purpose of this study was to use a nonhuman primate model to evaluate Vasalgel™, a high molecular weight polymer being developed as a contraceptive device for men.

**Methods:**

Sixteen adult male rhesus monkeys received intravas injections of Vasalgel, consisting of 25% styrene maleic acid in dimethyl sulfoxide. After a one-week recovery, males were returned to outdoor group housing, which included at least 3 and up to 9 intact, breeding females with a successful reproductive history.

**Results:**

Treated males have had no conceptions since Vasalgel injections. All males were housed with intact females for at least one breeding season and seven have been almost continually housed with females for 2 years. Complications were minor and included one incident of incorrect placement of Vasalgel into the vas deferens and the development of a sperm granuloma in one animal. Three unilateral vasectomies were performed, one was necessary for incorrect placement of Vasalgel, the other two were elective.

**Conclusions:**

Intravas injection of Vasalgel in sexually mature adult male rhesus monkeys was effective in preventing conception in a free-living, group environment. Complications were few and similar to those associated with traditional vasectomy.

## Résumé

**Contexte:**

Les possibilités de contraception masculine sont limitées. L’objectif de la présente étude était d’utiliser un modèle primate non humain pour évaluer le Vasalgel™, un polymère de haut poids moléculaire développé comme dispositif contraceptif pour homme.

**Matériel et méthodes:**

Seize singes rhésus mâles adultes ont eu une injection intradéférentielle de Vasalgel™ constitué d’une solution à 25% d’acide styrène-alt-maléique dans du diméthylsulfoxyde. Après une semaine de récupération, les mâles sont ramenés dans le groupe hébergé en plein air constitué d’au moins 3 et jusqu’à 9 femelles reproductrices normales de fertilité prouvée.

**Résultats:**

Les mâles traités n’ont induit aucune grossesse après l’injection de Vasalgel™. Les mâles ont tous été hébergés avec des femelles normales au moins pendant une saison reproductive, et sept d’entre eux ont quasiment été continuellement hébergés avec les femelles pendant 2 ans. Les complications furent mineures; elles ont été constituées par un incident de mise en place défectueuse du Vasalgel™ dans le canal déférent, et par le développement d’un granulome spermatique chez un animal. Trois vasectomies unilatérales ont été réalisées, l’une rendue nécessaire pour mise en place incorrecte du Vasalgel™, les deux autres le furent par choix.

**Conclusions:**

L’injection intradéférentielle de Vasalgel™ chez des singes rhésus mâles adultes s’est avérée efficace à empêcher toute conception dans un environnement de groupe vivant en liberté. Il y eut peu de complications; elles furent semblables à celles observées pour la vasectomie traditionnelle.

## Background

Options for non-permanent male contraceptives are limited to condoms, with no long-term methods currently available. While vasectomy is very effective as a permanent contraceptive, it is technically challenging to reverse, with low rates of fertility following vasectomy reversal surgery [1].

However, there have been recent advances in developing potentially reversible contraceptive methods that target the vas deferens. RISUG® (Reversible Inhibition of Sperm Under Guidance) is a styrene maleic anhydride (SMA) product for intra-vas injection that lines the vas, but does not occlude it [2]. The presence of RISUG in the vas is said to create a pH level that generates a positive charge that in turn disrupts the acrosomal membrane and prevents normal sperm transport through the female reproductive tract [3]. That product has been studied in nonhuman primates and found to be an effective contraceptive that could be removed from primates with percussive and vibratory massage, returning fertility [4, 5]. While various formulations of RISUG have been studied in India over several decades, no product has successfully been brought to market as of yet.

Another intra-vas injection product, Vasalgel™ is a high molecular weight polymer being developed in the US as a contraceptive device for men [6]. Vasalgel is an SMA acid polymer dissolved in DMSO (dimethyl sulfoxide), formulated to adhere to strict regulatory standards. Unlike RISUG, Vasalgel does not claim any pharmaceutical effect and is understood to work by occluding the vasa deferentia. After injection into the vas deferens, the SMA acid forms a hydrogel that appears to be tissue adherent, fills the lumen and acts as a mechanical barrier to the passage of sperm [6]. Vasalgel has proven to produce reliable, durable azoospermia in rabbits in which semen parameters were followed for 12 months after treatment [6]. Vasalgel was subsequently flushed from the vasa deferentia of these rabbits, successfully restoring sperm flow (Personal communication, Waller, 2016).

The purpose of the current study is to further test Vasalgel in a nonhuman primate model in a setting that is closer to a free-living environment, which includes access to intact, breeding females with a successful reproductive history. This study evaluates the effectiveness of Vasalgel in adult male rhesus monkeys living in small group housing for at least one breeding season and up to 2 years.

## Methods

### Vasalgel test group

Sixteen adult male rhesus monkeys (*Macaca mulatta*) were housed at the California National Primate Research Center (CNPRC) in large outdoor enclosures in groups of approximately 10 to 30 animals that included infants, juveniles, and adults. Ten of the 16 males had previous conceptions (Table 1). Males on this study were of varying social ranks with approximately equal numbers of very high ranking and low ranking animals. These housing units, referred to as “corn cribs”, are open air, dual-room gazebo-style housing, approx. 700 sq. ft. and include perches, play structures, chew toys and other enrichment devices. The adult population of each enclosure is typically one to three sexually mature males and two to nine sexually mature, intact females. Rhesus males and females reach sexual maturity at 3 to 4 years of age. The CNPRC is accredited by the Association for the Assessment and Accreditation of Laboratory Animal Care (AAALAC), associated with the University of California – Davis and is one of eight National Primate Research Centers maintained and funded by the National Institutes of Health. At the time of study enrollment, animals were assigned to the CNPRC outdoor breeding colony. All experimental procedures were approved in advance by the University of California, Davis Institutional Animal Care and Use Committee (IACUC). Animals were fed commercial monkey diet and water *ad libitum* plus daily foraging mix and weekly produce. Males enrolled were sexually mature, and ranged in age from 4 to 16 years (mean 7.08 ± 3.0 years) and weighed from 7 to 20 kg (mean 11.37 ± 3.20 kg). Males were intermittently housed indoors for procedure recovery and occasional treatment for routine health issues, but those periods were typically brief (1 to 2 days) and none exceed 20 days per episode. Indoor housing conditions were maintained at 37 °C with a 12:12 h light:dark cycle and provided with *ad libitum* monkey chow and water.

**Table 1:** Animal information on date of Vasalgel administration, age at time of Vasalgel administration, time males spent with intact female(s) after post-operative recovery through June 1, 2016, and previous conception number

Animal ID	Treatment date	Age at surgery	Dates housed with intact females	Total time with female	Previous conception number
1^a^	7/9/14	12 y 1 m	7/24/14 –3/2/2016	1 y 8 m	18
2^a^	7/9/14	8 y 3 m	8/4 – 12/21/14, 12/30/14–1/20/15, 2/2–11/3/15, 11/23/15– 3/2/2016, 3/10/16–5/23/16	1 y 8 m	5
3^a^	7/9/14	6 y 2 m	8/1/14 – 11/23/15, 12/15/15–2/24/16, 2/25– 3/2/2016	1 y 6 m 7 d	0
4^a^	7/9/14	5 y 9 m	7/18/14–1/2/15, 1/22–3/4/15, 3/10/15–4/17/15	8 m 2 d	1
5^a^	7/10/14	5 y 9 m	7/18–11/18/14, 11/26–12/10/14, 12/19/14–1/20/15, 1/23–3/4/15, 3/10/15–3/20/16	18 m 15 d	0
6^a^	7/10/14	8 y 2 m	7/15/14– 7/27/16	2 y 12 d	6
7^a^	7/10/14	16 y 3 m	7/15/14– 7/27/16	2 y 12 d	4
8	7/10/14	6 y 2 m	7/15/14–12/28/15	1 y 5 m 13 d	1
9	7/10/14	5 y 2 m	7/15/14–8/15/15, 8/25/15–5/2/2016	1 y 9 m 7 d	3
10	8/13/14	6 y 4 m	8/22–10/5/14, 10/14/-12/1/14, 12/4/14–1/12/15, 1/13/15–5/11/15	8 m 6 d	7
11	8/13/14	6 y 5 m	8/22/14–5/11/15	8 m 19 d	2
12	8/13/14	6 y 4 m	8/22/14–5/11/15	8 m 19 d	5
13	8/21/14	6 y 1 m	8/28/14–8/26/15	11 m 29 d	0
14	8/21/14	4 y 4 m	8/28–10/26/14, 10/28–12/4/14, 12/16/14–2/19/15, 2/27–4/17/15	6 m 28 d	0
15	8/29/14	4 y 4 m	9/9–12/2/14, 12/23/14–2/8/15, 2/18–4/17/15	5 m 14 d	0
16	8/29/14	6 y 1 m	9/9/14–4/17/15	7 m 8 d	0

*Y* year, *m* month, *d* day.

^a^Indicates males housed with females for two breeding seasons

### Age-matched vasectomy group

Only for the purpose of comparing the rate of post-procedure complications, 16 age-matched, previously vasectomized males were computer-selected from over 120 vasectomized males in the CNPRC colony. These males were randomly selected based on matching their age at vasectomy to the ages of the Vasalgel group males (mean 7.1 ± 3.0 years). No other comparisons were made. These males were not co-housed with the Vasalgel males.

### Test article

Vasalgel consisted of 25% solution by weight of styrene maleic acid (SMA) in DMSO. The average molecular weight (Mw) of the SMA anhydride (Poly(Styrene-co-Maleic Anhydride, CAS Registry Number: 25736-61-2) was 377 kDa according to standardized gel permeation chromatography (GPC) methodology (Scientific Polymer, Ontario, NY). The final test article was prepared and packaged in a nitrogen atmosphere in 2 ml glass vials by Polysciences, Inc. (Warrington, PA, USA).

### Implantation of test article

Animals were sedated with ketamine (10 mg/kg IM, Ketamine hydrochloride, Bioniche Pharma, Rosemont, IL) and atropine (0.05 mg/kg IM, Baxter HealthCare Corp. Deerfield, IL), weighed, intubated and then maintained on isoflurane (0.5–2.0%) for the duration of the surgery. After preparation of the surgical site, an approximate 2–3 cm incision was made just caudal to the right and left inguinal rings to expose the spermatic cords. The tunic was incised (~2 cm) and the vas deferens was isolated. The external diameter vas deferens in the rhesus monkey is approximately 2.0 mm [7], only slightly smaller than human [8]. The vasa deferentia were carefully elevated using stay suture and blunt instrumentation to stabilize the vas during injection. Once sufficiently isolated, the vas deferens was injected with approximately 100 μl of Vasalgel over about 30–45 s using a 24 gauge 3/4 inch catheter (Surflo, Terumo Medical Corporation, Somerset, NJ) inserted in a cranial direction.. A luer-lock syringe was required to maintain connection with the catheter as significant pressure was required to move the viscous Vasalgel through the catheter and into the vas deferens. After Vasalgel placement, the catheter was removed, and the vas deferens was carefully observed for about 30 s to assess for any leakage of Vasalgel. This volume of Vasalgel filled approximately 2 cm of the length of the vas deferens. The vas deferens was returned to the spermatic cord and the site was closed with 3-0 Vicryl ((Ehticon Inc., Sommerville, NJ) in a simple continuous pattern. Animals received buprenorphine hydrochloride (0.03 mg/kg IM, Buprenex Reckitt Benckiser Pharmaceuticals Inc., Richmond, VA) twice a day for 1 day and ketoprofen (5 mg/kg IM, Ketofen, Fort Dodge, Fort Dodge, IA) once a day for 3 days for post-operative analgesia. Animals also received cefazolin (25 mg/kg IM, ANCEF ® GlaxoSmithKline, Research Triangle Park, NC) twice a day for 3 days. All surgical procedures were performed by the same surgical team and animal recovery was uneventful. Animals were monitored post-operatively daily for 7 days and then returned to group housing. Standard veterinary care was provided throughout the study.

### Fertility assessment

Animals in corn crib housing units are sedated at least twice per year for routine health monitoring, preventive veterinary care and for pregnancy assessment via palpation or ultrasound. Paternity of all offspring is determined via blood samples collected from offspring at approximately 4 to 6 months of age and submitted for genetic testing to the Veterinary Genetics Laboratory, School of Veterinary Medicine, University of California, Davis [9]. The expected pregnancy rate for sexually mature females in “corn crib” style housing is approximately 80% per breeding season, from unpublished data kept for colony management purposes over the past 40 years.

## Results

Intravas injection of the Vasalgel was successfully accomplished in all 16 male subjects. All males on this study were housed with females for at least one breeding season, which for rhesus monkeys in the Northern Hemisphere is generally mid-September to mid-February [10]. However, our experience at CNPRC timed mating colony is that females can become pregnant from mid-September to mid-May. Rhesus females have menstrual cycles of approximately 28 days and have cycles most of the year with the exception of a few months in summer – generally June, July and August, thus males had extensive exposure to cycling females. The mean time (± SEM) of co-housing with females was 1.19 ± 0.14 years with a range of 5 months 14 days (0.45 years) to 2 years. The shortest duration began in early September and continued until mid-April (Male # 15), thus covering a complete breeding season. No conceptions occurred for any of the Vasalgel males. As detailed in Table 1, seven of the males (Male # 1 – 7) have been almost continually housed with intact females for two breeding seasons without conceptions. There were no systematic behavioral studies performed, but routine behavioral monitoring had observation of breeding behavior for all study males that were monitored.

### Instillation of Vasalgel

Due to the viscous nature of Vasalgel, a significant amount of pressure was required to instill material into the narrow vas deferens. The use of a luer-lock syringe to prevent separation of the catheter from the syringe was required and application of a consistent level of pressure over 10 to 20 s proved to be the most successful method. Efficiency of application improved with surgeon experience handling the material and effectively manipulating the vas deferens to allow for smooth catheter placement.

### Surgical complications

#### Incorrect placement

(Animal 3) During instillation of Vasalgel, the injection site of the left vas deferens appeared to have significant leakage. It was noted that there was potential damage to the wall of the vas deferens associated with infiltration of Vasalgel under the sheath. Due to the uncertainty of successful Vasalgel placement, a traditional vasectomy was performed on the side in question. The affected area of the left vas deferens was isolated, double ligated with Vicryl 3–0 and transected. Closure of the surgical site was the same as for Vasalgel sites. Vasalgel was successfully placed into the right vas deferens in the animal and no complications were observed.

#### Sperm granuloma

(Animal 10) During a routine sedation approximately 2 months post-operatively, a small (approximate 1 cm) opening near the right surgical site was observed. The associated tissue was erythematous and a mild amount of white, viscous discharge was present near the opening. It was determined cytologically to be a sperm granuloma and a surgical repair, resulting in a traditional vasectomy as described above, was performed on the right side. No further complications were observed.

#### Social trauma-surgical intervention

**Fig. 1:**
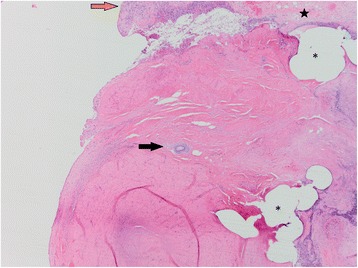
Animal 2: Abundant collagenous stroma surrounded by pyogranulomatous inflammation (*orange arrow*), and fibrin (*black star)*. The clear spaces represent areas that contained sutures (*asterisk*). In the center of the image, is a small globule of basophilic to amphophilic material (*black arrow*, presumed Vasalgel). H&E, 40x

**Fig. 2:**
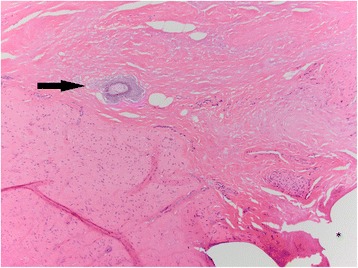
Animal 2: Same section as Fig. 1. In the center of the image, is a small, irregular globule of basophilic to amphophilic material (*black arrow*, presumed Vasalgel). There is no associated inflammation. Clear areas of sutures are noted (*asterisk*). H&E, 100x

Animal 2 was admitted to the CNPRC indoor hospital for nonspecific trauma associated with bite wounds and lacerations to the right leg, left cheek and neck, 1 day after returning to his home cage, which was 7 daysays after Vasalgel placement. Examination revealed a firm swelling palpable at the left surgical site, extending into the scrotum. It could not be determined if the swelling was associated with trauma from fighting with other males, a sperm granuloma or a hydrocele. Although no external evidence of trauma to the genital area was found at the time of the physical examination, possible internal trauma from male-to-male aggression could not be definitely ruled out. Exploratory surgery was elected, resulting in an attempt to perform a traditional vasectomy as described above (using 4–0 Prolene) due to the presence of a hydrocele. The presumed vas deferens was submitted for histopathology. The histopathology revealed that the spermatic cord had multiple areas of pyogranulomatous inflammation admixed with free spermatids, fibrin and hemorrhage along the surface of the spermatic cord, confirming the presence of a left spermatic granuloma (Fig. 1). Adjacent to these areas, but not associated with any inflammation, were small irregular foci of basophilic to amphophilic granular to crystalline material presumed to be Vasalgel (Fig. 2). Thus, it appeared that the inflammation was associated with the presence of extraluminal sperm, rather than from the presence of Vasalgel. After reviewing the histopathology and medical report, it was determined that the presence of Vasalgel was incidental to the post-operative complications.

To determine the comparative complication rate between Vasalgel placement and traditional vasectomy, 16 age-matched vasectomized adult rhesus monkeys were identified. As shown in Table 2, five out of the 32 vasa (15.6%) from traditionally vasectomized animals developed sperm granulomas.

**Table 2:** Complications encountered in aged-matched control rhesus macaques undergoing traditional vasectomy at the CNPRC

Animal ID	Age at surgery	Complication
1	12 y 4 m	None
2	8 y 3 m	None
3	6 y 2 m	None
4	5 y 9 m	Left sperm granuloma
5	5 y 8 m	None
6	8 y 2 m	Mild left testicular atrophy
7	16 y 3 m	Left sperm granuloma
8	6 y 2 m	Right sperm granuloma
9	5 y 2 m	Left sperm granuloma
10	6 y 4 m	None
11	6 y 4 m	Mild suture reaction
12	6 y 4 m	None
13	6y 2 m	None
14	4 y 4 m	None
15	4 y 4 m	None
16	6 y 2 m	Left side sperm granuloma

*Y* year, *m* month

## Discussion

Vasalgel placement into the vas deferens of mature male rhesus macaque monkeys produced reliable contraception, as evidenced by the lack of pregnancies in sexually mature, reproductively viable females with which the study males were co-housed. Vasalgel placement allowed for a rapid return to group housing, providing minimal disruption to the social structure of the group while not impeding natural behaviors such as mating. Furthermore, as evidenced in the histology from Animal 2, it appears that the presence of Vasalgel does not incite a localized inflammatory reaction. This finding is similar to that found in the rabbit, in which tissue response to the presence of Vasalgel was minimal [6]. Although further histologic evidence from rhesus macaques would be helpful, the goal of this study was to rapidly return animals to their home environments to allow them to continue to maintain their social status within their group hierarchy.

Further support for the tissue compatibility of SMA products is evidenced by reports on the use of RISUG in similar animal models. The safety of the SMA vas-occlusive product RISUG in nonhuman primates has been previously reported [5, 11]. Long term studies in langur monkeys indicated that after 540 days of vas occlusion and 150 days of non-surgical reversal, the impact on associated tissues was minimal and temporary. For example, the vas deferens, which had initially showed loss of epithelial cells and loosened smooth muscle layer, evidenced a return to normal within 150 days of reversal. The epididymis, prostate and seminal vesicles remained unaltered and no sperm granulomas or sperm antibodies were found in the study subjects [5]. Thus, previous research on RISUG supports the efficacy and safety of SMA intra-vas contraception.

Complications during Vasalgel placement in one animal (Animal 3) were associated with damage to the wall of the vas deferens, likely as a result of incorrect placement of the catheter, resulting in incomplete penetration of the wall of the vas deferens. This resulted in extraluminal leakage of Vasalgel within the thin fibrous sheath surrounding the vas, likely weakening the overall structure. Once pressure was applied to fill the vas, deficits within the wall were evident, requiring a traditional vasectomy on this side. Regardless, the occurrence rate of surgical complication was low (one out of 32 vasa deferentia) and occurred very early in the study, during the first day of Vasalgel instillation in one of the first animals. Subsequent surgical dates proceeded without incident, likely a result of surgeon experience gained with Vasalgel instillation, improving ease of successful placement.

In this study, we noted only one sperm granuloma associated with the Vasalgel placement post-operatively. A sperm granuloma is a collection of extravasated sperm that is observed near the vasa deferentia in vasectomized patients, generally caused by leakage from the incision in the vas deferens or from a rupture of the epididymis. Sperm granulomas can present as a small, pea-sized lump under the skin or as a leakage of white, viscous material from the skin incision site. Sperm granulomas are common complications of vasectomy in humans, typically occurring in the 2nd to 3rd week post-operatively in approximately 60% of cases [12]. Most sperm granulomas are asymptomatic and self-resolve over time. From our results in this study, Vasalgel instillation appears to have a low rate of sperm granuloma formulation (1/32 vas deferens), and may have a lower rate than traditional vasectomy in rhesus macaques when compared to other studies [13, 14]. Aged matched control rhesus macaques from the CNPRC that had a traditional vasectomy were found to have a higher incidence of sperm granuloma than animals that underwent the Vasalgel procedure (Table 2). The high rate of sperm granulomas after traditional vasectomy in sexually mature rhesus monkeys may be associated with both physical and sexual activity after release into their enclosures, as physical and sexual activity is typically restricted in men after vasectomy procedures in an attempt to reduce the occurrence of sperm granulomas.

The only other complication observed was in Animal 2 and may have been a consequence of conspecific trauma. Mature males are known to fight at increased rates during the breeding season [15, 16] and, due to their large canine teeth, can inflict severe wounds in a single, brief altercation. Although conspecific trauma is a risk associated with group housing, group housing allows for development of the normal social hierarchy and interactions that this species may experience in the wild [17], provides opportunities for engagement with animals of varying ages, encourages self-motivated exercise through freedom of movement within the enclosure and is considered imperative to the psychological well-being of non-human primates [18].

## Conclusion

Vasalgel placement within the vas deferens seems to be an effective method for contraception in adult male rhesus macaques living in social group settings. Placement prevented conception and was durable throughout the observation period in all animals, which covered as long as two full breeding seasons in some animals. Additionally, the presence of Vasalgel appears to be well tolerated and placement resulted in minimal complications. Further study, including the possibility of reversal by flushing the gel from the vasa deferentia in this species, is warranted.

## Abbreviations

CNPRC: California national primate research center
DMSO: Dimethyl sulfoxide
GPC: Permeation chromatography
IACUC: Institutional animal care and use committee
RISUG®: Reversible inhibition of sperm under guidance
SMA: Styrene maleic anhydride
